# fcfdr: an R package to leverage continuous and binary functional genomic data in GWAS

**DOI:** 10.1186/s12859-022-04838-0

**Published:** 2022-07-30

**Authors:** Anna Hutchinson, James Liley, Chris Wallace

**Affiliations:** 1 MRC Biostatistics Unit, University of Cambridge, Cambridge, UK; 2 MRC Human Genetics Unit, University of Edinburgh, Edinburgh, UK; 3 The Alan Turing Institute, London, UK; 4 Cambridge Institute of Therapeutic Immunology and Infectious Disease (CITIID), University of Cambridge, Cambridge, UK; 5 Department of Medicine, University of Cambridge, Cambridge, UK

**Keywords:** GWAS, Functional genomics, Power, FDR, Multiple testing

## Abstract

**Background:**

Genome-wide association studies (GWAS) are limited in power to detect associations that exceed the stringent genome-wide significance threshold. This limitation can be alleviated by leveraging relevant auxiliary data, such as functional genomic data. Frameworks utilising the conditional false discovery rate have been developed for this purpose, and have been shown to increase power for GWAS discovery whilst controlling the false discovery rate. However, the methods are currently only applicable for continuous auxiliary data and cannot be used to leverage auxiliary data with a binary representation, such as whether SNPs are synonymous or non-synonymous, or whether they reside in regions of the genome with specific activity states.

**Results:**

We describe an extension to the cFDR framework for binary auxiliary data, called “Binary cFDR”. We demonstrate FDR control of our method using detailed simulations, and show that Binary cFDR performs better than a comparator method in terms of sensitivity and FDR control. We introduce an all-encompassing user-oriented CRAN R package (https://annahutch.github.io/fcfdr/; https://cran.r-project.org/web/packages/fcfdr/index.html) and demonstrate its utility in an application to type 1 diabetes, where we identify additional genetic associations.

**Conclusions:**

Our all-encompassing R package, fcfdr, serves as a comprehensive toolkit to unite GWAS and functional genomic data in order to increase statistical power to detect genetic associations.

**Supplementary Information:**

The online version contains supplementary material available at 10.1186/s12859-022-04838-0.

## Background

A stringent significance threshold is required to identify robust genetic associations in genome-wide association studies (GWAS) due to multiple testing constraints. Leveraging relevant auxiliary data, such as functional genomic data, has the potential to boost statistical power in order to detect associations that exceed the stringent significance threshold.

The conditional false discovery rate (cFDR) is a Bayesian FDR measure that additionally conditions on auxiliary data to call significant associations. Let $p_1,\ldots ,p_m \in (0,1]$ be a set of *p* values corresponding to the null hypotheses of no association between SNPs $1,\ldots ,m$ and a trait of interest (denoted by $H_0$). Let $q_1,\ldots ,q_m$ be auxiliary data values corresponding to the same SNPs. Assume that *p* and *q* are realisations of random variables *P*, *Q* satisfying:1The cFDR is then defined as the probability that a random SNP is null for the trait given that the observed *p* values and auxiliary data values at that SNP are less than or equal to values *p* and *q* respectively [1, 2]. That is,

2 $$\begin{aligned} cFDR(p,q)=Pr(H_0|P\le p, Q\le q). \end{aligned}$$

It should be noted that, although the Bayes-optimal decision quantity $Pr(H_0|P=p, Q=q)$ [3, 4] is asymptotically more powerful for hypothesis testing, it is practically more difficult to estimate accurately in finite-sample settings [5].

The cFDR approach was originally developed to leverage GWAS *p* values from related traits, thereby exploiting genetic pleiotropy to increase GWAS discovery [1, 2, 6]; however, these early methods failed to control the FDR. Consequently, Liley and Wallace [5] developed an extension to the cFDR approach that transforms cFDR estimates into “*v*-values” which are analogous to *p* values and can therefore be used to control FDR (for example in the Benjamini–Hochberg procedure [7]).

Motivated by the enrichment of GWAS SNPs in particular functional genomic annotations [8], Flexible cFDR was developed to extend the usage of the cFDR approach to the accelerating field of functional genomics [9]. Several related methods exist for multiple testing in the presence of auxiliary information [4, 10–12], but Flexible cFDR has been shown to outperform these methods in terms of usability, versatility, accessibility and FDR control [9]. Nonetheless, a disadvantage of Flexible cFDR is that it cannot be used to leverage auxiliary data with a binary representation, such as whether SNPs are synonymous or non-synonymous, or whether they reside in regions of the genome with specific activity states.

Here we present an extension to the cFDR approach that supports binary auxiliary data, called Binary cFDR. In a simulation-based analysis, we compare the performance of Binary cFDR to that of an existing approach, Boca and Leek’s FDR regression [13], which has been shown to outperform other methods in terms of FDR control, power, applicability and consistency of results by an independent research group [14]. We introduce a cFDR toolbox in the form of an R package (https://github.com/annahutch/fcfdr) that supports various auxiliary data types and which is available on CRAN (https://cran.r-project.org/web/packages/fcfdr/index.html). Finally, we demonstrate the utility of our methods and software by iteratively leveraging three distinct types of relevant auxiliary data with GWAS *p* values for type 1 diabetes to uncover additional genetic associations.

## Implementation

### The cFDR framework

We begin by describing the standard cFDR framework. Bayes theorem and standard probability rules are used to derive:

3 $$\begin{aligned} \begin{aligned} cFDR(p,q)&=Pr(H_0|P\le p, Q\le q) \\&= \dfrac{Pr(P\le p|H_0,Q\le q)\times Pr(H_0|Q\le q)}{Pr(P\le p|Q\le q)} \\&= \dfrac{Pr(P\le p|H_0,Q\le q) \times Pr(Q\le q|H_0)Pr(H_0)}{Pr(P\le p, Q\le q)}. \end{aligned} \end{aligned}$$

To construct a conservative estimator of the cFDR, approximate $Pr(P\le p|H_0,Q\le q)\approx p$ (from property (1); note that if property (1) holds and *P* is correctly calibrated then this approximation is an equality) and $Pr(H_0)\approx 1$ (since associations are rare in GWAS):4where $\widehat{ }$ is used to denote that these are estimates under the assumption  . The methods used to estimate the cumulative densities in equation (4) vary across approaches. For example, in the original cFDR approach they are estimated using empirical cumulative density functions [1, 5, 15] whilst in Flexible cFDR they are estimated using kernel density estimation [9].

However, the  values do not directly control the FDR [15]. Instead, a method proposed by Liley and Wallace [5] can be used to generate *v*-values, which are essentially the probability of a newly-sampled realisation (*p*, *q*) of *P*, *Q* attaining an as extreme or more extreme  value than that observed, given $H_0$. The *v*-values are therefore analogous to *p* values and can be used in any conventional error-controlling multiple testing procedure. The derivation of *v*-values also allows for the method to be applied iteratively to incorporate additional layers of auxiliary data.

### Extension for binary covariate data

We introduce an extension to the cFDR framework that permits binary covariate data, and call our method “Binary cFDR”.

As before, let $p_1,\ldots ,p_m \in (0,1]$ be a set of *p* values corresponding to the null hypotheses of no association between the SNP and the trait of interest. Now, let $q_1,\ldots ,q_m \in \{0,1\}$ be a set of binary covariates for the same *m* SNPs. Denote the null (no association) and alternative (association) hypotheses as $H_0$ and $H_1$ respectively and assume that *p* and *q* are realisations of random variables *P*, *Q* satisfying property (1). We follow the standard methodology introduced by Liley and Wallace [5] to derive a *v*-value, $v_i$, for each $(p_i,q_i)$ pair.

Since all *q* are binary, the support of *P*, *Q* is two lines $(0,1) \times \{0,1\}$. We consider rejection regions of the form $L(p_0, p_1) = (P\le p_0, Q=0) \cup (P\le p_1, Q=1),$ where $p_0$ and $p_1$ are to be determined.

We wish to find *v*-values such that for all $\alpha$,

5 $$\begin{aligned} \begin{aligned} Pr(v_i<\alpha |H_0)&=\alpha \\ Pr(v_i<\alpha |H_1)&\text { is maximal.} \end{aligned} \end{aligned}$$

That is, the *v*-values behave like typical *p* values in that they are uniform under the null, but are as small as possible under the alternative hypothesis. Appendix A.1 in [5] (and also [16] and [17], for example) show that this corresponds to rejection regions formed by the set of points for which $f_0(p,q)/f_1(p,q) < k(\alpha )$, for some *k*, where $f_0(p,q)=f(P=p, Q=q|H_0)$ and $f_1(p,q)=f(P=p, Q=q|H_1)$. If $f_1(p,q)$ is non-increasing in *p*, then such optimal rejection regions are of the type $L(p_0,p_1)$ defined above (we describe behaviour in other cases in Additional File 1). That is, $p_0$ and $p_1$ will satisfy the property

6 $$\begin{aligned} \dfrac{f_0(p_0,0)}{f_1(p_0,0)}=\dfrac{f_0(p_1,1)}{f_1(p_1,1)}. \end{aligned}$$

Let

7 $$\begin{aligned} f(p,q)=f(P=p, Q=q)=\pi _0 f_0(p,q)+(1-\pi _0) f_1(p,q), \end{aligned}$$

where $\pi _0=Pr(H_0)$. Then equation (6) implies that

8 $$\begin{aligned} \dfrac{f(p_1,1)}{f_0(p_1,1)} = \dfrac{f(p_0,0)}{f_0(p_0,0)}. \end{aligned}$$

To solve equation (6) for $p_0$ and $p_1$, we approximate

9 $$\begin{aligned} \dfrac{f_0(p_i,q_i)}{f(p_i,q_i)}&= \dfrac{Pr(P=p_i, Q=q_i|H_0)}{Pr(P= p_i, Q=q_i)} \end{aligned}$$

10 $$\begin{aligned}{} &\approx \dfrac{Pr(P\le p_i, Q=q_i|H_0)}{Pr(P\le p_i, Q=q_i)} \end{aligned}$$

11 $$\begin{aligned}{} &= \dfrac{Pr(P\le p_i| Q=q_i, H_0)Pr(Q=q_i|H_0)}{Pr(P\le p_i|Q=q_i)Pr(Q=q_i)} \end{aligned}$$

12where  and *m* is the number of SNPs. Approximation (10) is discussed in Additional File 1. If $q_i=0$ then we set $p_0=p_i$ and use approximation (12) to solve equation (6) for $p_1$. If $q_i=1$, then we set $p_1=p_i$ and solve for $p_0$. In practise, we do this using a fold-removal protocol for estimation to ensure that rejection rules are not applied to the same data on which those rules were determined. Specifically, we leave out each chromosome in turn and use the remaining SNPs to estimate the values for the held out SNPs.

We derive the final *v*-values by integrating the distribution of *P*, *Q* under the null hypothesis over the rejection regions:

13 $$\begin{aligned} \int _{L(p_0,p_1)}df_0&= Pr((P,Q) \in L(p_0, p_1)|H_0) \end{aligned}$$

14 $$\begin{aligned}{} &= Pr((P\le p_0, Q=0) \cup (P\le p_1, Q=1) | H_0) \end{aligned}$$

15 $$\begin{aligned}{} &\begin{aligned}{} &= Pr(P\le p_0, Q=0|H_0) \\&\qquad + Pr(P\le p_1, Q=1|H_0) \end{aligned} \end{aligned}$$

16 $$\begin{aligned}{} &\begin{aligned}{} &= Pr(P\le p_0|Q=0, H_0)Pr(Q=0|H_0) \\&\qquad + Pr(P\le p_1|Q=1, H_0)Pr(Q=1|H_0) \end{aligned} \end{aligned}$$

17 $$\begin{aligned}{} &= p_0\times (1-q_0) + p_1\times q_0 \end{aligned}$$

where  .

The *v*-value, $v_i$, can be interpreted as the probability that a randomly-chosen (*p*, *q*) pair has a more extreme cFDR value than cFDR($p_i,q_i$) under $H_0$. That is, a quantity analogous to a *p* value. This means that, as in the original cFDR approach [5], the Binary cFDR method can be applied iteratively to incorporate additional layers of auxiliary data, whereby the *v*-values from the previous iteration are used as the principal trait p values in the current iteration. The derivation of *v*-values analogous to *p* values also means that they can be readily FDR controlled using any FDR controlling procedure that allows for slightly dependent *p* values (as in GWAS), such as the Benjamini–Hochberg procedure [5].

### fcfdr R package

We have created a CRAN R package, fcfdr, that implements the Flexible cFDR and Binary cFDR approaches (https://cran.r-project.org/web/packages/fcfdr/index.html). Our recently updated package supports a wide range of auxiliary data types and is particularly suited to leveraging functional genomic data with GWAS test statistics, as explored below and also in several fully reproducible vignettes that are available on the package web-page (https://annahutch.github.io/fcfdr/).

## Results

### Simulation based analysis

We evaluated the performance of Binary cFDR as implemented in the fcfdr R package using a simulation-based analysis. In each simulation, we applied Binary cFDR iteratively 5 times to represent leveraging multi-dimensional binary covariates. We additionally compared our results to those when using a comparator method, Boca and Leek’s FDR regression (BL) [13], which has been shown to outperform other methods by an independent research group [14].

**Fig. 1:**
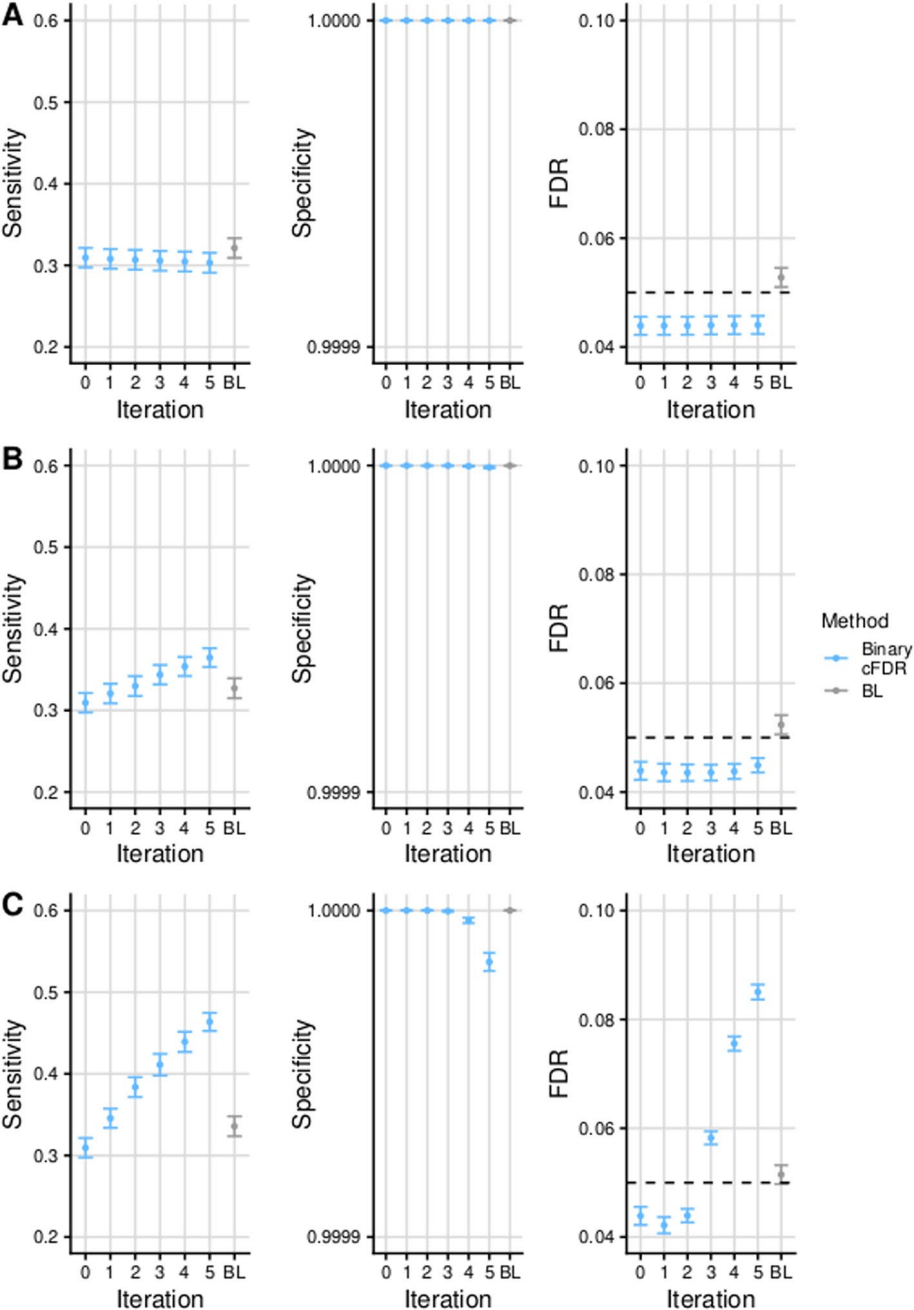
Simulation results for Binary cFDR and BL. Mean +/− standard error for the sensitivity, specificity and FDR of FDR values (derived from the Benjamini–Hochberg procedure) from Binary cFDR when iterating over independent (**A**; “simulation A”) and dependent (**B**; “simulation B” and **C**; “simulation C”) binary auxiliary data. BL refers to results when using Boca and Leek’s FDR regression to leverage the 5-dimensional covariate data. Iteration 0 corresponds to the original FDR values. Results were averaged across 100 simulations

We expect that leveraging irrelevant data should not change our conclusions about a study. Figure 1A shows that the sensitivity and specificity remain stable across iterations and that the FDR was controlled at a pre-defined level when using Binary cFDR to leverage independent binary auxiliary data with arbitrary GWAS *p* values. In contrast, when leveraging relevant data we hope that the sensitivity improves whilst the specificity remains high, which is what we observed for Binary cFDR in Fig. 1B.

It is known that the cFDR approach should not be used to iterate over correlated auxiliary data that is capturing the same functional mark, as SNPs with a modest *p* but extreme *q* will incorrectly attain greater significance with each iteration (for a more detailed explanation see [9]). Our final set of simulations involved iterating over correlated auxiliary data values (mean Pearson correlation coefficient = 0.3) that capture the same “functional mark” (80% of functional SNPs were expected to have an auxiliary data value of 1 in each iteration). The lack of FDR control in these sets of simulations (Fig. 1C) serves as a salutary reminder that care should be taken not to repeatedly iterate over functional data that is capturing the same genomic feature.

When bench-marking the performance of Binary cFDR against that of BL, we found that BL was consistently less powerful than Binary cFDR when leveraging dependent auxiliary data (Fig. 1B, C). In contrast, BL was more powerful than Binary cFDR when leveraging independent auxiliary data, but this was at the cost of a marginal loss of FDR control (Fig. 1A). In fact, the FDR control of BL was similar across all simulations, even when using correlated auxiliary data in Fig. 1C.

### Application to type 1 diabetes

**Fig. 2:**
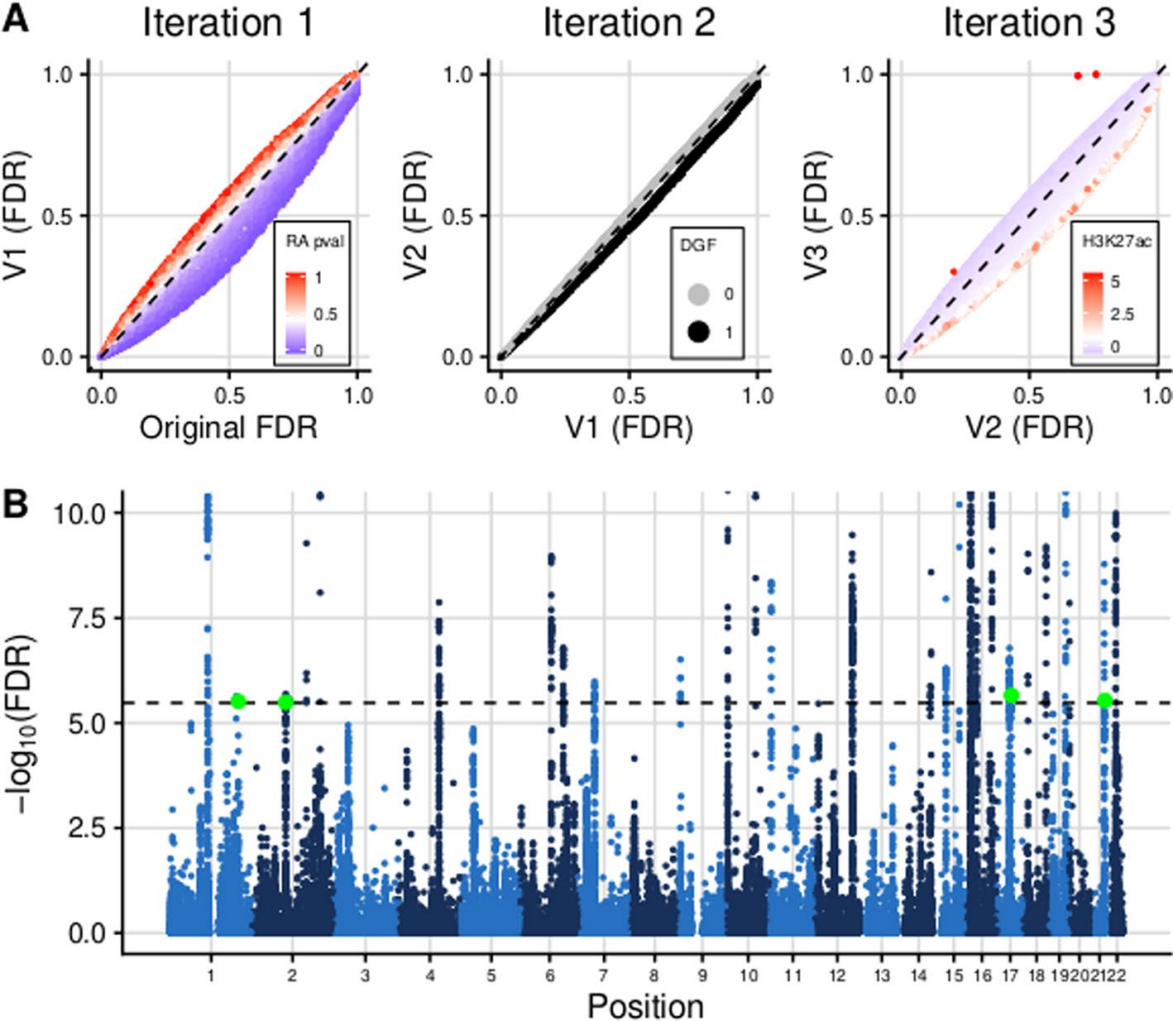
Summary of cFDR results from type 1 diabetes application. **A** FDR values (derived from the Benjamini–Hochberg procedure) before and after each iteration of cFDR, coloured by the auxiliary data values. **B** Manhattan plot of ($-log_{10}$) FDR values (*y*-axis truncated to aid visualisation). Green points indicate the four lead variants that were newly FDR significant after cFDR. Black dashed line at FDR significance threshold ($FDR=3.3\times 10^{-6}$)

We demonstrate the utility of fcfdr in an application to type 1 diabetes which is fully reproducible (https://annahutch.github.io/fcfdr/articles/t1d_app.html). Using *p* values from an Immunochip study of type 1 diabetes [18] as our primary data set, we iteratively leveraged *p* values from an Immunochip study of a related immune-mediated trait (rheumatoid arthritis; RA), binary data measuring SNP overlap with regulatory factor binding sites and enhancer-associated H3K27ac ChIP-seq data in cell types relevant to type 1 diabetes (Fig. 2).

Our method identified 101 SNPs as newly FDR significant ($FDR\le 3.3\times 10^{-6}$ which corresponds to $p\le 5\times 10^{-8}$; see Methods). These SNPs had relatively small *p* values for RA (median $p=0.007$ compared with median $p=0.422$ in full data set), were more likely to be found in regulatory factor binding sites (40.6% in binding sites compared to 23.4% in full data set) and had larger H3K27ac fold change values in relevant cell types (median value was 1.44 compared with 0.576 in full data set). In contrast, 45 SNPs that were significant in the original GWAS data set became not significant after applying cFDR, and these had relatively high *p* values for RA (median $p=0.620$), were less likely to be found in regulatory factor binding sites (4.4% in binding sites) and had smaller H3K27ac fold change values (median value was 0.431).

**Table 1:** Table of newly significant index SNPs from type 1 diabetes application

rsID	Position	Ref/Alt	OR	SE	*p* value	*v*-value	RA *p* value	DGF	H3K27ac percentile	Gene
rs1052553	chr17:44073889	A/G	0.889	0.022	$8.16\times 10^{-8}$	$3.10\times 10^{-8}$	$6.76\times 10^{-3}$	1	2.2th	*STH*
rs3024505	chr1:206939904	G/A	0.864	0.027	$6.39\times 10^{-8}$	$4.51\times 10^{-8}$	0.601	1	87.4th	*IL19*
rs6518350	chr21:45621817	A/G	0.880	0.024	$9.64\times 10^{-8}$	$4.26\times 10^{-8}$	0.062	0	72.7th	*ICOSLG*
rs13415583	chr2:100764087	T/G	0.904	0.019	$1.06\times 10^{-7}$	$4.81\times 10^{-8}$	$1.91\times 10^{-6}$	0	14.4th	*AFF3*

For each of the four newly significant SNPs from the cFDR analysis, we list the rsID, genomic position (hg19; Position), reference and alternative alleles (Ref/Alt), odds ratio (OR), standard error (SE) and *p* value reported in the primary GWAS data set [18], *v*-value from the cFDR analysis, GWAS *p* value for rheumatoid arthritis [27] (RA *p* value), binary indicator of SNP overlap with regulatory factor binding sites (DGF), percentile of mean H3K27ac fold change value across asthma relevant cell types (H3K27ac percentile) and the closest protein-coding gene

The original GWAS identified 38 significant genomic regions (based on our definition of genomic regions; see Methods). All of these were found to be significant in the cFDR analysis, which additionally identified 4 genomic regions with index SNPs that became newly significant (Table 1). When using a larger Immunochip study of type 1 diabetes for validation [19] (see Methods) we found that three out of the four lead variants were present and that these had smaller *p* values in the validation GWAS data set than the discovery GWAS data set: rs1052553 validation $p=1.65\times 10^{-15}$, rs3024505 validation $p=9.13\times 10^{-14}$ and rs13415583 validation $p=4.76\times 10^{-9}$.

When using BL to leverage the same auxiliary data, only 46 SNPs were identified as newly FDR significant, and these had larger *p* values for RA (median $p=0.1942$), were less likely to be found in regulatory factor binding sites (32.6% in binding sites) and had similar H3K27ac fold change values (median value was 1.48) compared with the 101 SNPs identified as newly significant in the cFDR analysis. At the locus level, BL only identified 1 newly significant index SNP, rs3024505, which was also identified by cFDR. No SNPs that were significant in the original GWAS data set became not significant after applying BL.

## Conclusions

We have described Binary cFDR, a novel implementation of the cFDR approach that supports binary auxiliary data. Binary cFDR controls the FDR and increases sensitivity where appropriate, and outperforms an existing method in terms of sensitivity and FDR control. Binary cFDR is implemented in an all-encompassing CRAN R package, fcfdr, that can be used to implement the cFDR approach for a wide variety of auxiliary data types. We have demonstrated the versatility of our software in an application to type 1 diabetes, whereby we incorporated both binary and continuous auxiliary data simultaneously to uncover additional genetic associations that were replicated in a larger study.

## Methods

### Simulation analysis

#### Simulating GWAS results (p)

Following Hutchinson et al. (2021) [9], we first simulated GWAS *p* values for the arbitrary “principal trait”. We collected haplotype data for 3781 individuals from the UK10K project (REL-2012-06-02) [20] at 80,356 SNPs residing on chromosome 22 with $MAF \ge 0.05$ (to match the convention that genetic association studies identify common genetic variation). We split the haplotype data into 24 LD blocks representing approximately independent genomic regions defined by the LD detect method [21]. We then further stratified these so that no more than 1000 SNPs were present in each block, subsequently recording the LD block that each SNP resided in.

We used the simGWAS R package (https://github.com/chr1swallace/simGWAS) [22] to simulate *Z*-scores for SNPs within each block. The simulate_z_scores function in thee simGWAS R package requires input for (i) the number of cases and controls (ii) the causal variants (iii) the log odds ratios at the causal variants and (iv) haplotype frequencies. For our simulation analysis, we selected 5000 cases and 5000 control samples, and within each block we randomly sampled 2, 3 or 4 causal variants with log OR effect sizes simulated from the standard Gaussian prior used in case-control genetic fine-mapping studies, $N(0,0.2^2)$ [23]. For the haplotype frequency parameter, we supplied a data.frame of haplotypes using the UK10K data, with a column of computed frequencies for each haplotype. We collated the *Z*-scores from each region and converted these to *p* values representing the evidence of association between the SNPs and the arbitrary principal trait.

#### Simulating auxiliary data (q)

We considered three use-cases of Binary cFDR (simulations A-C) defined by dependence on the principal trait *p* value ($p_i$) and correlations between realisations of *q*. In simulation A we leveraged binary auxiliary data that was independent of $p_i$: $q_i\sim \text {Bernoulli}(0.05)$. In simulations B and C we leveraged binary auxiliary data that was dependent on $p_i$ by first defining “functional SNPs” as causal variants plus any SNPs within 10,000-bp (to incorporate SNPs residing in the same arbitrary “functional mark”), and “non-functional SNPs” as the remainder. We then sampled $q_i$ from different distributions for functional and non-functional SNPs. Specifically, in simulation B we sampled:

18 $$\begin{aligned} \begin{aligned} q_i\sim {\left\{ \begin{array}{ll} \text {Bernoulli}(0.05), \text { if SNP { i} is non-functional} \\ \text {Bernoulli}(0.4), \text { if SNP { i} is functional.} \\ \end{array}\right. } \end{aligned} \end{aligned}$$

Our method will likely be used to leverage functional genomic data iteratively, and so we also evaluated the impact of repeatedly iterating over auxiliary data that captured the same functional mark. Thus, in simulation C we iterated over realisations of *q* that were highly correlated:

19 $$\begin{aligned} \begin{aligned} q_i\sim {\left\{ \begin{array}{ll} \text {Bernoulli}(0.05), \text { if SNP { i} is non-functional} \\ \text {Bernoulli}(0.8), \text { if SNP { i} is functional.} \\ \end{array}\right. } \end{aligned} \end{aligned}$$

Note that the auxiliary data is correlated in simulation C because the functional SNPs are the same across iterations in each simulation.

#### Implementing Binary cFDR and BL

We used the fcfdr::binary_cfdr function to implement Binary cFDR in our simulation analysis. To avoid overfitting we used a leave-one-out procedure, whereby the LD block [21] was used as the group variable. In each simulation for each simulation scenario, we applied Binary cFDR iteratively 5 times to represent leveraging multi-dimensional covariates.

To implement BL, we used the lm_qvalue function in the swfdr Bioconductor R package (version 1.16.0) [24], using a covariate matrix that consisted of five columns for the auxiliary data values to derive adjusted *p* values.

#### Evaluating sensitivity, specificity and FDR control

To quantify the results from our simulations, we used the Benjamini–Hochberg procedure to derive FDR-adjusted *v*-values from Flexible cFDR, which we call “FDR values” for conciseness (that is, we used the stats::p.adjust R function with method=“BH”). We then calculated proxies for the sensitivity (true positive rate) and the specificity (true negative rate) at an FDR threshold of $\alpha = 5\times 10^{-6}$, which roughly corresponds to the genome-wide significance *p* value threshold of $5\times 10^{-8}$ (the maximum FDR value amongst SNPs with raw *p* value $\le \times 10^{-8}$ was $5.4\times 10^{-6}$). We defined a subset of “truly associated SNPs” as any SNPs with $r^2\ge 0.8$ with any of the causal variants. Similarly, we defined a subset of “truly not-associated SNPs” as any SNPs with $r^2\le 0.01$ with all of the causal variants. (Note that there are 3 non-overlapping sets of SNPs: “truly associated”, “truly not-associated” and neither of these). We calculated the sensitivity proxy as the proportion of truly associated SNPs that were called significant and the specificity proxy as the proportion of truly not-associated SNPs that were called not significant.

To assess whether the FDR was controlled within a manageable number of simulations, we raised $\alpha$ to 0.05 and calculated the proportion of SNPs that were called FDR significant but were truly not-associated (that is, $r^2\le 0.01$ with all of the simulated causal variants).

### Application to type 1 diabetes

#### GWAS data

We downloaded full harmonised GWAS summary statistics for type 1 diabetes [18] from the NHGRI-EBI GWAS Catalog [25] (study GCST005536 accessed on 08/10/21) and used these as the principal trait *p* values. This data was for 6670 European type 1 diabetes cases and 12,262 European controls. We used the LDAK software (https://dougspeed.com/ldak/) to obtain LDAK weights for each SNP, and defined our independent SNP set (used to fit the KDE in Flexible cFDR) as the set of SNPs given a non-zero LDAK weight (an LDAK weight of 0 means that its signal is (almost) perfectly captured by neighbouring SNPs). We used MAFs estimated from the CEU sub-population samples in the 1000 Genomes Project Phase 3 data set [26], and for any SNPs with missing MAF we randomly sampled a value from the empirical distribution of non-missing MAFs.

To define independent loci for our locus-level results, we first calculated LD between each pair of SNPs using haplotype data from the 503 individuals of European ancestry in the 1000 Genomes Project Phase 3 data set [26]. We then used PLINK’s LD-clumping algorithm with a 5-Mb window and an $r^2$ threshold of 0.01. This conservative clumping approach sorts SNPs into ascending order of *p* value and then moves down the list, sequentially removing SNPs within a 5-Mb window and with $r^2>0.01$. The SNP with the smallest *p* value in the data set in each LD clump was called the “lead variant”.

#### Validation GWAS data set

We downloaded full harmonised GWAS summary statistics for type 1 diabetes [19] from the NHGRI-EBI GWAS Catalog [25] (study GCST90013445 accessed on 08/10/21) and used this as our validation GWAS data set. The samples in the discovery GWAS data set [18] were a subset of those in the validation data set, and so we said that a discovery validated if it’s corresponding *p* value was smaller in [19] than [18]. The validation data set was for 16,159 European type 1 diabetes cases and 25,386 European controls.

#### Auxiliary data

We downloaded full harmonised GWAS summary statistics for rheumatoid arthritis (RA) [27] from the NHGRI-EBI GWAS Catalog [25] (study GCST005569 accessed on 08/10/21). We mapped each SNP in the type 1 diabetes GWAS data set to its corresponding *p* value for RA using genomic coordinates and rsIDs. We removed 6044 SNPs from the analysis which did not have a corresponding *p* value for RA.

We downloaded SNP-level annotations for all 1000 Genomes SNPs from the baseline-LD model (version 2.2) described in [28]. We extracted values for the binary annotation “DGF_ENCODE” which quantifies sites of transcription factor occupancy. Briefly, this annotation is derived from merging all DNase I digital genomic footprinting (DGF) regions from the narrow-peak classifications across 57 cell types [29, 30]. DGF regions (corresponding to DGF annotation values of 1) are expected to precisely map sites where regulatory factors bind to the genome [31]. We matched each SNP in the type 1 diabetes GWAS data set to its binary DGF annotation using genomic coordinates. We removed 2811 SNPs from the analysis that did not have a corresponding DGF annotation value.

We downloaded consolidated fold-enrichment ratios of H3K27ac ChIP-seq counts relative to expected background counts from NIH Roadmap Epigenomics Mapping Consortium [32] in nine primary tissues and cells relevant for type 1 diabetes (CD3, CD4+ CD25int CD127+ Tmem, CD4+ CD25+ CD127- Treg, CD4+ CD25- Th, CD4+ CD25- CD45RA+, CD4 memory, CD4 naive, CD8 memory, CD8 naive). Specifically, we downloaded the bigWig files, converted these to wig files and then to bed files, and then mapped each SNP in the type 1 diabetes GWAS data set to its corresponding genomic region in the bed files and recorded the H3K27ac fold change values in each cell type using the bedtools intersect utility. For SNPs on the boundary of a genomic region (and therefore mapping to two regions) we randomly selected one of the regions. We observed that the fold change values across relevant cell types were highly correlated ($r>0.65$) and therefore averaged values across cell types to avoid iterating over highly correlated auxiliary data that is likely capturing the same functional mark. We transformed the averaged fold change values ($q:=log(q+1)$) to deal with long tails.

#### Implementation

We used the fcfdr::flexible_cfdr and fcfdr::binary_cfdr functions to leverage the auxiliary data with type 1 diabetes GWAS *p* values iteratively. We used the chromosome for which each SNP resided for the group parameter in fcfdr::binary_cfdr, and we used the estimated MAF values for the optional maf parameter in the fcfdr::flexible_cfdr function. We used the stats::p.adjust function with method=“BH” to derive FDR values from the *v*-values (after the 3 iterations) and used these as the output of interest. We used an FDR threshold of $FDR\le 3.3\times 10^{-6}$ to call significant SNPs, which corresponded to the genome-wide significance threshold $p\le 5\times 10^{-8}$ (it was the maximum FDR value amongst SNPs with raw *p* values $\le 5\times 10^{-8}$ in the discovery GWAS data set). The full data and code to replicate the analysis are available from https://annahutch.github.io/fcfdr/articles/t1d_app.html.

## Supplementary information

**Additional file 1.** Further details on Binary cFDR methodology. Further details on Binary cFDR methodology, including an overview and comments on the key assumptions.

**Additional file 2.** Supplementary results from T1D application. Supplementary results from T1D application quantifying the relationship between the “principal p-values” (p) and the auxiliary data (q) in each iteration of the T1D application.

## Data Availability

The datasets analysed during the current study are available in the github repository: https://github.com/annahutch/fcfdr.* Availability and requirements*: Project name: fcfdr Project home page: https://annahutch.github.io/fcfdr/ Operating system(s): Tested on Linux, MacOS and Windows Programming language: R Other requirements: R ($\ge 3.5.0$) License: MIT license Any restrictions to use by non-academics: None.
