# Supplementary material for: fcfdr: an R package to leverage continuous and binary functional genomic data in GWAS
Source: BMC Bioinformatics. 2022 Jul 30;23:310. doi: 10.1186/s12859-022-04838-0 (PMC9338519; doi:10.1186/s12859-022-04838-0)
Supplement: Supplementary file 1 — Additional file 1. Further details on Binary cFDR methodology. Further details on Binary cFDR methodology, including an overview and comments on the key assumptions. [file 12859_2022_4838_MOESM1_ESM.pdf]

Additional File 1

Further details on Binary cFDR methodology

1 Overview

Let $p_1, ..., p_m \in (0,1]$ be a set of p-values corresponding to the null hypotheses of no association between the SNP and the trait of interest. Let $q_1, ..., q_m \in \{0,1\}$ be a set of binary covariates for the same m SNPs, and denote the null (no association) and alternative (association) hypotheses as H_0 and H_1 respectively. Assume that p and q are realisations of random variables P, Q satisfying:

$$\begin{aligned} (P|H_0) &\sim U(0,1) \\ P &\perp\!\!\!\perp Q|H_0. \end{aligned} \tag{1}$$

We follow the standard methodology introduced by Liley and Wallace (2021) to derive a v-value, v_i, for each (p_i, q_i) pair. That is, we find the smallest rejection region that each observation (p_i, q_i) is contained in, estimate the distribution of P, Q under the null hypothesis and integrate this distribution over the rejection region to obtain the v-value.

Since all q are binary, the support of P, Q is two lines and so the rejection regions are of the form

$$L(p_0, p_1) = (P \leq p_0, Q = 0) \cup (P \leq p_1, Q = 1), \tag{2}$$

where p_0 and p_1 are unknown.

We wish to find v-values such that for all α,

$$\begin{aligned} &Pr(v_i < \alpha|H_0) = \alpha \\ &Pr(v_i < \alpha|H_1) \text{ is maximal.} \end{aligned} \tag{3}$$

That is, the v-values behave like p-value in that they are uniform under the null, but are as small as possible under the alternative hypothesis. Appendix A.1 in Liley and Wallace (2021) (and also Du and Zhang (2014) and Alishahi *et al.* (2016), for example) show that this corresponds to rejection regions formed by the set of points for which $f_0(p,q)/f_1(p,q) < k(\alpha)$, for some k, where $f_0(p,q) = f(P = p, Q = q|H_0)$ and $f_1(p,q) = f(P = p, Q = q|H_1)$. That is, p_0 and p_1 will satisfy the property

$$\frac{f_0(p_0,0)}{f_1(p_0,0)} = \frac{f_0(p_1,1)}{f_1(p_1,1)}. \tag{4}$$

Let

$$f(p,q) = f(P = p, Q = q) = \pi_0 f_0(p,q) + (1-\pi_0) f_1(p,q), \tag{5}$$

where $\pi_0 = Pr(H_0)$. Then equation (4) implies

$$f_0(p_0,0) f_1(p_1,1) = f_0(p_1,1) f_1(p_0,0) \tag{6}$$

$$f_0(p_0,0)\frac{f(p_1,1) - \pi_0 f_0(p_1,1)}{1-\pi_0} = f_0(p_1,1)\frac{f(p_0,0) - \pi_0 f_0(p_0,0)}{1-\pi_0} \tag{7}$$

$$\frac{f(p_1,1) - \pi_0 f_0(p_1,1)}{f_0(p_1,1)} = \frac{f(p_0,0) - \pi_0 f_0(p_0,0)}{f_0(p_0,0)} \tag{8}$$

$$\frac{f(p_1,1)}{f_0(p_1,1)} = \frac{f(p_0,0)}{f_0(p_0,0)}. \tag{9}$$

To solve equation (4) for p_0 and p_1, we approximate

$$\frac{f_0(p_i,q_i)}{f(p_i,q_i)} = \frac{Pr(P = p_i, Q = q_i|H_0)}{Pr(P = p_i, Q = q_i)} \tag{10}$$

$$\approx \frac{Pr(P \leq p_i, Q = q_i|H_0)}{Pr(P \leq p_i, Q = q_i)} \tag{11}$$

$$= \frac{Pr(P \leq p_i|Q = q_i, H_0) Pr(Q = q_i|H_0)}{Pr(P \leq p_i|Q = q_i) Pr(Q = q_i)} \tag{12}$$

$$\approx \frac{p_i \times \widehat{Pr(Q = q_i|H_0)}}{|j : p_j \leq p_i, q_j = q_i|/m} \tag{13}$$

where $\widehat{Pr(Q=q_i|H_0)} = \frac{|j : q_j = q_i, p_j > 1/2|}{|j : p_j > 1/2|}$ and m is the total number of observations (i.e. the total number of SNPs). If $q_i = 0$ then we set $p_0 = p_i$ and use approximation (13) to solve equation (4) for p_1. If $q_i = 1$, then we set $p_1 = p_i$ and solve for p_0.

Specifically, if $q_i = 0$ then we set $p_0 = p_i$ and solve the following for p_1:

$$\frac{p_i \times \frac{|j : q_j = 0, p_j > 1/2|}{|j : p_j > 1/2|}}{|j : p_j \leq p_i, q_j = 0|/m} = \frac{p_1 \times \frac{|j : q_j = 1, p_j > 1/2|}{|j : p_j > 1/2|}}{|j : p_j \leq p_1, q_j = 1|/m} \tag{14}$$

$$\frac{p_i \times \frac{|j : q_j = 0, p_j > 1/2|}{|j : p_j > 1/2|}}{|j : p_j \leq p_i, q_j = 0| \times \frac{|j : q_j = 1, p_j > 1/2|}{|j : p_j > 1/2|}} = \frac{p_1}{|j : p_j \leq p_1, q_j = 1|}. \tag{15}$$

In practise, we do this using a fold-removal protocol for estimation to ensure that rejection rules are not applied to the same data on which those rules were determined. Specifically, we either leave out each chromosome or each LD block in turn and use the remaining SNPs to estimate the values for the held out SNPs.

Similarly, if $q_i = 1$, then we set $p_1 = p_i$ and solve the following for p_0:

$$\frac{p_0 \times \frac{|j : q_j = 0, p_j > 1/2|}{|j : p_j > 1/2|}}{|j : p_j \leq p_0, q_j = 0|/m} = \frac{p_i \times \frac{|j : q_j = 1, p_j > 1/2|}{|j : p_j > 1/2|}}{|j : p_j \leq p_i, q_j = 1|/m} \tag{16}$$

$$\frac{p_0}{|j : p_j \leq p_0, q_j = 0|} = \frac{p_i \times \frac{|j : q_j = 1, p_j > 1/2|}{|j : p_j > 1/2|}}{|j : p_j \leq p_i, q_j = 1| \times \frac{|j : q_j = 0, p_j > 1/2|}{|j : p_j > 1/2|}}. \tag{17}$$

We derive the final v-values by integrating the distribution of P,Q under the null hypothesis over the rejection regions:

$$\begin{aligned}
\int_{L(p_0,p_1)} df_0 &= Pr((P,Q) \in L(p_0,p_1)|H_0) && (18)\\
&= Pr((P \leq p_0, Q = 0) \cup (P \leq p_1, Q = 1)|H_0) && (19)\\
&= Pr(P \leq p_0, Q = 0|H_0) \\
&\quad + Pr(P \leq p_1, Q = 1|H_0) && (20)\\
&= Pr(P \leq p_0|Q = 0, H_0)Pr(Q = 0|H_0) \\
&\quad + Pr(P \leq p_1|Q = 1, H_0)Pr(Q = 1|H_0) && (21)\\
&= p_0 \times (1 - q_0) + p_1 \times q_0 && (22)
\end{aligned}$$

where $q_0 = \widehat{Pr(Q = 1|H_0)}$.

The v-value, v_i, can be interpreted as the probability that a randomly-chosen (p,q) pair has a more extreme cFDR value than cFDR(p_i,q_i) under H_0. That is, a quantity analogous to a p-value that can be readily FDR controlled using any FDR controlling procedure that allows for slightly dependent p-values, such as the Benjamini-Hochberg (BH) procedure (Benjamini and Hochberg, 1995).

2 Non-monotonicity of p-value distributions under H_1

In this section we consider the distributions of the random variables

$$f_1(p,1) = Pr(P = p, Q = 1|H_1)$$
$$f_1(p,0) = Pr(P = p, Q = 0|H_1)$$

Recalling that optimal rejection regions (in the sense of regions R which maximise $Pr(P \in R|H_1)$ for fixed $Pr(P \in R|H_0)$) are sets of p,q defined by $f_0(p,q)/f(p,q) \leq \alpha$ for some α, it is clear if both $f_1(p,1)$ and $f_1(p,0)$ are non-increasing in p, then optimal rejection regions will be of the form $L(p_0,p_1)$ as specified in the main manuscript (that is, $L(p_0,p_1) = (P \leq p_0, Q = 0) \cup (P \leq p_1, Q = 1))$ for some p_0,p_1.

In general, if a test is well designed and sensitive to H_1, p-values are expected to be non-decreasing in p, suggesting that $f_1(p,1)$ and $f_1(p,0)$ will generally be at least roughly decreasing over most of $(0,1)$. If we allow the densities $f_1(p,1)$, $f_1(p,0)$ to be arbitrary, then optimal rejection regions may similarly be arbitrary pairs of Borel subsets of $(0,1)$. The optimal rejection region of type $L(p_0,p_1)$ is not readily specifiable in this case. However, in such settings, the p-values themselves are of little use, as 'small' p-values are not useful in distinguishing H_0 and H_1.

It is possible that real-world p-value densities may have non-decreasing forms; for instance, $f_1(p,1) \sim \beta\left(\frac{1}{2},\frac{1}{2}\right)$, for which the optimal rejection region will include intervals $[0,p_1),(1-p_1,1]$. We demonstrate that our approach of taking a rejection region as $L(p_0,p_1)$ where we choose p_0,p_1 to approximately satisfy $f_0(p,1)/f(p,1) = f_0(p,0)/f(p,0)$ is still reasonable in this setting. We will assume that random variables associated with p-values have smooth densities on $(0,1)$.

2.1 Locally decreasing distributions

We firstly note that if values p_0, p_1 exist such that

$$\frac{f_0(p_1,1)}{f(p_1,1)} = \frac{f_0(p_0,0)}{f(p_0,0)} \tag{23}$$

$$\left.\frac{\partial}{\partial p} f_1(p,1)\right|_{p=p_1} \leq 0 \tag{24}$$

$$\left.\frac{\partial}{\partial p} f_1(p,0)\right|_{p=p_0} \leq 0 \tag{25}$$

then $L(p_0,p_1)$ will be at least a local optimum, in the sense that small variations in p_0, p_1 which conserve the type-1 error rate $Pr\left((p,q) \in L(p_0,p_1)|H_0\right)$ cannot improve the power: $Pr\left((p,q) \in L(p_0,p_1)|H_1\right)$. To see this, suppose that type-1 error rate is conserved at α, so:

$$\begin{aligned}
\alpha &= Pr\left((P,Q) \in L(p_0,p_1)|H_0\right) \\
&= Pr(P \leq p_0|Q=0,H_0)Pr(Q=0|H_0) + Pr(P \leq p_1|Q=1,H_0)Pr(Q=1|H_0) \\
&= p_0 Pr(Q=0|H_0) + p_1 Pr(Q=1|H_0)
\end{aligned}$$

so with type-1 error rate α, we have

$$p_1 = \frac{1}{Pr(Q=1|H_0)}\left(\alpha - p_0 Pr(Q=0|H_0)\right)$$

and the change in power with p_0 is

$$\begin{aligned}
\frac{\partial}{\partial p_0} Pr((p,q) \in L(p_0,p_1)|H_1) &= \frac{\partial}{\partial p_0}\left(\int_0^{p_0} f_1(p,0)dp \right.\\
&\quad \left. + \int_0^{p_1} f_1(p,1)dp\right)\\
&= f_1(p_0,0) - f_1(p_1,1)\frac{Pr(Q=0|H_0)}{Pr(Q=1|H_0)}\\
&\propto \frac{f_1(p_0,0)}{Pr(Q=0|H_0)} - \frac{f_1(p_1,1)}{Pr(Q=1|H_0)}\\
&= \frac{f_1(p_0,0)}{f_0(p_0,0)} - \frac{f_1(p_1,1)}{f_0(p_1,1)}\\
&= 0
\end{aligned}$$

and indeed since $\frac{\partial}{\partial p_0} f_1(p_1,1) > 0$, we have

$$\begin{aligned}
\frac{\partial^2}{\partial p_0^2} Pr((p,q) \in L(p_0,p_1)|H_1) &= \frac{\partial}{\partial p_0} f_1(p_0,0) - \frac{\partial}{\partial p_0} f_1(p_1,1)\frac{Pr(Q=0|H_0)}{Pr(Q=1|H_0)}\\
&= \frac{\partial}{\partial p_0} f_1(p_0,0) + \frac{\partial}{\partial p_0} f_1(p_1,1)\frac{Pr(Q=0|H_0)}{Pr(Q=1|H_0)}\\
&< 0
\end{aligned}$$

so $L(p_0,p_1)$ is locally optimal.

An immediate implication is that forms of $f_0(p,1)$, $f_1(p,1)$ which are decreasing for low p and increasing for high p, a choice of p_0, p_1 satisfying conditions 23-25 will be the best possible such choice, given that rejection regions are constrained to the type $L(p_0,p_1)$.

3 Comment on approximation

In the cFDR approach, we approximate the ratio

$$\frac{f_1(p,q)}{f(p,q)} = \frac{f(P=p,Q=q|H_1)}{f(P=p,Q=q)} \approx \frac{Pr(P \leq p, Q=q|H_1)}{Pr(P \leq p, Q=q)}$$

or equivalently

$$\begin{aligned} \frac{f_1(p,q)}{f(p,q)} &\approx \frac{Pr(P \leq p, Q=q|H_1)}{Pr(P \leq p, Q=q)} \\ \Leftrightarrow \qquad f_1(p,q) &\approx \frac{1}{p} Pr(P \leq p, Q=q|H_1) \end{aligned} \tag{26}$$

In typical cases (in particular when $f_1(p,q) = \Theta(p^b)$ for some $b > 0$ with $\Theta()$ in the sense of $p \to 0$), this approximation is roughly true in the sense that:

$$\begin{aligned} \lim_{p \to 0} \log(LHS) = \lim_{p \to 0} \log(RHS) &= \infty \\ \lim_{p \to 0} \frac{\log(LHS)}{\log(RHS)} &= 1 \end{aligned}$$

In particular if $P \sim 2\Phi(-|Z|)$ with $Z \sim N(0,\sigma^2)$, then $\log(LHS) - \log(RHS) = O(1)$, where the $O(1)$ is typically small.

To see why approximation 26 often holds, we note it is equivalent to:

$$f_1(p,q) \approx \frac{1}{p} \int_0^p f_1(\rho,q) d\rho$$

If we have $f_1(p,q) = \Theta(p^b)$ for some $b > 0$ (as $p \to 0$), as is the case when P is derived from a Gaussian distribution as above, then we have

$$\frac{1}{p} \int_0^p f_1(\rho,q) d\rho = \frac{1}{b} \Theta(p^{b+1}) = \Theta(p^b)$$

and

$$\lim_{p \to 0} \frac{\log(LHS)}{\log(RHS)} = \lim_{p \to 0} \frac{b \log(p) + O(1)}{b \log(p) + O(1)} = 1$$

Approximation 26 is typically incorrect by a small factor dependent on the spread of the summary statistics from which p-values are derived (σ in the example above). In general this approximation is typically good enough. In particular, in the cFDR and GWAS settings, several factors contribute to its suitability:

1. GWAS p-value thresholds are typically small (c. 5×10^{-8}), so limiting approximations as $p \to 0$ are reasonable. In addition, changes to p-value thresholds by small factors (< 10) tend to make little difference to findings, so errors of small factors are tolerable.
2. Spread of summary statistics in GWAS tends to depend on underlying disease mechanisms and sample sizes, and hence will generally be fairly consistent with Q. Since the eventual aim of the approximation is to equate the sides of 26 when $q = 0$ and $q = 1$, this means that the errors will typically be in the same direction, and to some extent cancel out.
3. Because rejection thresholds are chosen at a later stage in the cFDR procedure (as a threshold on v-values), error in approximation 26 results only in a non-optimal trade-off between leniency of p-value thresholds when $q = 0$ or $q = 1$ rather than a direct overall loss of power.

We demonstrate a simple example. Suppose we have

$$\begin{aligned} Pr(Q = 0|H_0) &= \frac{1}{2}, & Pr(Q = 1|H_0) &= \frac{1}{2} \\ Pr(Q = 0|H_1) &= \frac{1}{100}, & Pr(Q = 1|H_1) &= \frac{99}{100} \end{aligned}$$

and

$$\begin{aligned} (P|Q = 0, H_1) &= 2\Phi(-|Z_0|); & Z_0 &\sim N(0, 6^2) \\ (P|Q = 1, H_1) &= 2\Phi(-|Z_1|); & Z_1 &\sim N(0, 7^2) \end{aligned}$$

These settings are reasonably typical of GWAS; the covariate value $Q = 1$ is fifty times more likely to be present in true associations, and corresponds to a modest change in distribution of p-values under H_1.

Given $p_0 = 5 \times 10^{-}8$, the value of p_1 corresponding to an optimal rejection region is

$$\begin{aligned} p_1 &= \left\{ p : \frac{f_1(p, 1)}{f(p, 1)} = \frac{f_0(p_0, 0)}{f_0(p_0, 0)} \right\} \\ &= 6.2 \times 10^{-6} \end{aligned}$$

and the value of p_1 given by our approximation is

$$p_1 = \left\{ p : \frac{Pr(P \leq p, Q = 1|H_1)}{Pr(P \leq p, Q = 1)} = \frac{Pr(P \leq p_0, Q = 0|H_1)}{Pr(P \leq p_0, Q = 0)} \right\}$$
$$= 7.1 \times 10^{-6}$$

which is a reasonable approximation.

References

Alishahi, K., Ehyaei, A. R., and Shojaie, A. (2016). A Generalized Benjamini-Hochberg Procedure for Multivariate Hypothesis Testing. *arXiv:1606.02386 [stat]*.

Benjamini, Y. and Hochberg, Y. (1995). Controlling the False Discovery Rate: A Practical and Powerful Approach to Multiple Testing. *Journal of the Royal Statistical Society. Series B (Methodological)*, **57**(1), 289–300.

Du, L. and Zhang, C. (2014). Single-index modulated multiple testing. *Annals of Statistics*, **42**(4), 1262–1311.

Liley, J. and Wallace, C. (2021). Accurate error control in high-dimensional association testing using conditional false discovery rates. *Biometrical Journal*.
