# Supplementary material for: fcfdr: an R package to leverage continuous and binary functional genomic data in GWAS
Source: BMC Bioinformatics. 2022 Jul 30;23:310. doi: 10.1186/s12859-022-04838-0 (PMC9338519; doi:10.1186/s12859-022-04838-0)
Supplement: Supplementary file 2 — Additional file 2. Supplementary results from T1D application. Supplementary results from T1D application quantifying the relationship between the “principal p-values” (p) and the auxiliary data (q) in each iteration of the T1D application. [file 12859_2022_4838_MOESM2_ESM.pdf]

Additional File 2

Supplementary results from T1D application

We evaluated the relationship between the "principal p-values" (p) and the auxiliary data (q) in each iteration. In iteration 1 the Pearson correlation coefficient between p (T1D GWAS p-values) and q (RA GWAS p-values) was 0.092. In iteration 2 the Pearson correlation coefficient between p (v-values from iteration 1) and q (binary DGF value) was -0.022. In iteration 3 the Pearson correlation coefficient between p (v-values from iteration 2) and q (log transformed average H3K27ac counts) was -0.083.

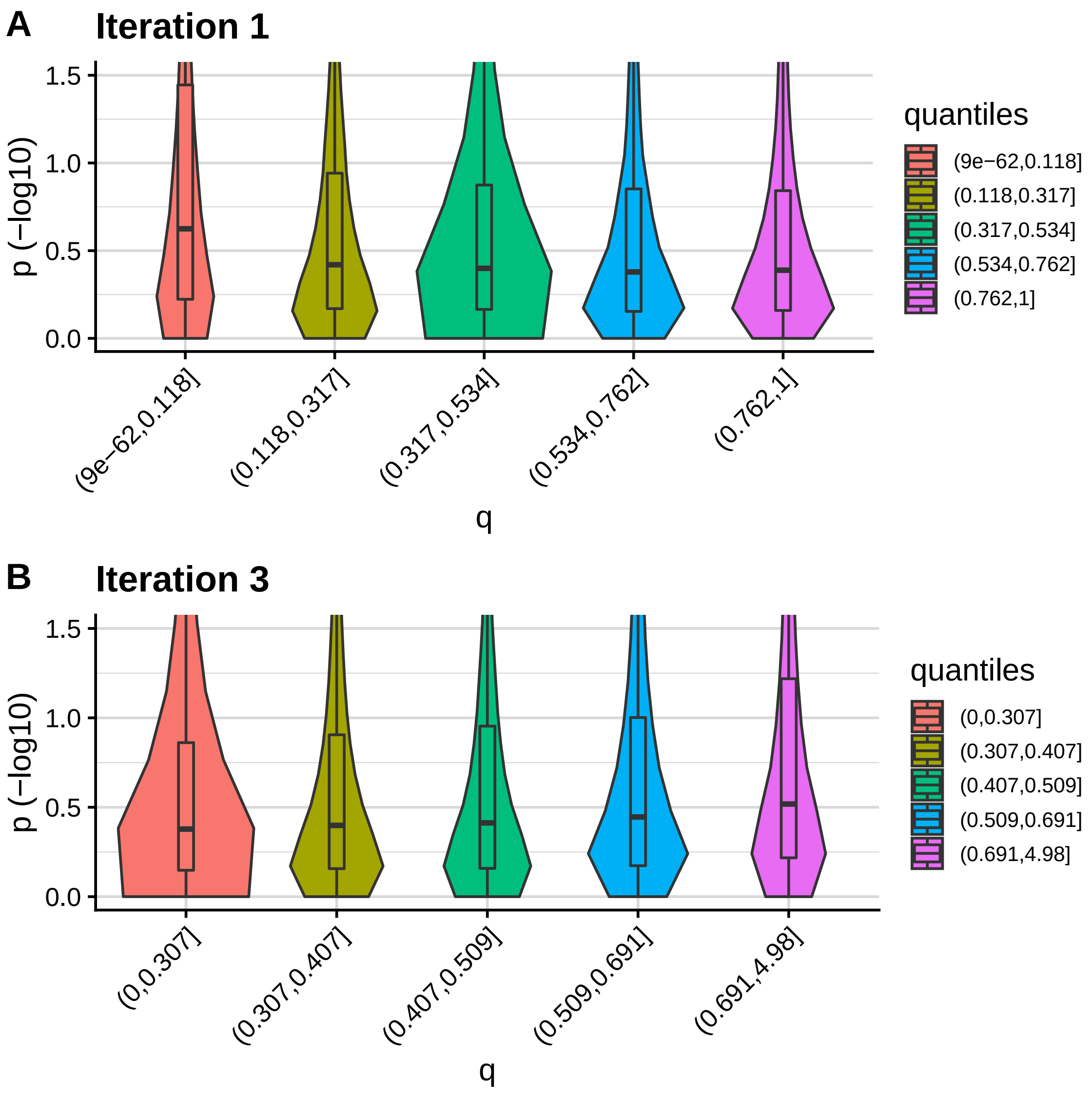

Additional File 2, Fig. 1: Violin plots showing the relationship between p and q in iterations 1 and 3 of the cFDR framework in the T1D application. Figure generated using the `fcfdr::corr_plot` function with default parameter values (`https://annahutch.github.io/fcfdr/reference/corr_plot.html`).
